# Treatment of Diptheria

**Published:** 1860-12

**Authors:** 


### TREATMENT OF DIPHTHERIA.

If there is much excitement in the beginning. I give an emetic of ipecac, and followed, if the bowels are constipated, with some mild cathartic, such as rhubarb. After the action of the emetic, if one is given, or otherwise immediately, I cauterize the diphtherite patches on the tonsils with the nitrate of silver, applying the stick direct. I think it much neater than the solution, and can be applied where you wish it, and there only. I load the end of a quill with the stick—the best caustic holder I know of, and always at hand in a country practice. With this I cauterize the throat once every twenty-four hours, so long as necessary. I make a saturated solution of the chlorate of potassa, to which, if the case requires it, I add quinine and Bourbon whisky.

℞ Chlorate of potassa, ℥j.
Sulph. quinine, grs. xvj.
Water, ℥xvj.

Dose of a teaspoonful to a tablespoonful every three hours, according to age. In cases where there is a pale, moist, cool skin, a frequent and soft pulse, the addition of the whisky to the above formula will prove of great benefit.

As a stimulating gargle I use—

℞ Apple vinegar, ℥ viij.

Capsicum, ʒ j.

Muriate of soda, ʒ j. M.

Use three or four times a day as a gargle. As an astringent gargle I use a decoction of oak or persimmon bark, cold.

As a styptic in hæmorrhage from the ulcerated surface of the throat, I used a strong solution of the sulphate of zinc and ice.

The external application that I used in all my cases was a piece of of old bacon bound to the throat. The bacon usually in from one to three days brings out a crop of pimples, producing a safe and valuable counter irritation. Owing to the tendency of diphtherite inflammation to attack abraded surfaces, I do not consider it judicious to use the ordinary blistering preparations.

This course of treatment in my hands has been successful in every case but one that I saw before the laryngial symptoms were set up. It has been adopted by other physicians with like success.

As a constitutional remedy, I rely upon the chlorate of potassa and quinine, with the additional stimulus, when required.

As a topical application, I rely upon the nitrate of silver, with the gargles above mentioned. In many cases I allow a liberal fluid diet, if it can be taken.—*Dr. Barbour.*

